# The Statistics of EEG Unipolar References: Derivations and Properties

**DOI:** 10.1007/s10548-019-00706-y

**Published:** 2019-04-10

**Authors:** Shiang Hu, Dezhong Yao, Maria L. Bringas-Vega, Yun Qin, Pedro A. Valdes-Sosa

**Affiliations:** 1 The Clinical Hospital of Chengdu Brain Science Institute, MOE Key Lab for NeuroInformation, University of Electronic Science and Technology of China, Chengdu, China; 2 Cuban Neuroscience Center, Havana, Cuba

**Keywords:** The family of unipolar references, Average reference, REST reference, Maximum likelihood estimate, No memory property

## Abstract

In this brief communication, which complements the EEG reference review (Yao et al. in Brain Topogr, 2019), we provide the mathematical derivations that show: (1) any EEG reference admits the general form of a linear transformation of the ideal multichannel EEG potentials with reference to infinity; (2) the average reference (AR), the reference electrode standardization technique (REST), and its regularized version (rREST) are solving the linear inverse problems that can be derived from both the maximum likelihood estimate (MLE) and the Bayesian theory; however, REST is based on more informative prior/constraint of volume conduction than that of AR; (3) we show for the first time that REST is also a unipolar reference (UR), allowing us to define a general family of URs with unified notations; (4) some notable properties of URs are ‘no memory’, ‘rank deficient by 1’, and ‘orthogonal projector centering’; (5) we also point out here, for the first time, that rREST provides the optimal interpolating function that can be used when the reference channel is missing or the ‘bad’ channels are rejected. The derivations and properties imply that: (a) any two URs can transform to each other and referencing with URs multiple times will not accumulate artifacts; (b) whatever URs the EEG data was previously transformed with, the minimum norm solution to the reference problem will be REST and AR with and without modeling volume conduction, respectively; (c) the MLE and the Bayesian theory show the theoretical optimality of REST. The advantages and limitations of AR and REST are discussed to guide readers for their proper use.

## Introduction

This brief communication provides the detailed mathematical demonstrations as well as some new findings that complement the EEG reference review (Yao et al. 2019). That review provides an overview of the state of the art of proposals for the EEG reference. When discussing current issues, it was evidently necessary to summarize the novel statistical approaches to the reference problem which is best described as the estimation of the potential at infinity. We thus build the family of unipolar references (URs), derive a novel maximum likelihood estimator (MLE), formulate a few notable properties and compare them to the related Bayesian estimators for this linear inverse problem first described in (Hu et al. 2018c).

### The Origin of EEG Reference Electrode Problem

The reference electrode and all the active electrodes over the scalp can record the linearly superimposed activities from all the neural sources. This means that the reference signal recorded by the reference electrode is correlated with the signals recorded by the active electrodes. Although an infinity reference is practically impossible, a lead field with the infinity reference is mathematically obtainable. By the quasi-static approximation of the Maxwell equation (Gulrajani 1998), the EEG potential ${\varvec{\upvarphi }}$ with the infinity reference related to the neural source currents ${\mathbf{j}}$ is

1 $$ {\varvec{\upvarphi }} = {\mathbf{K}}_{\infty } {\mathbf{j}} $$

Here ${\mathbf{K}}_{\infty }$, known as the lead field matrix, expresses the forward model computed with the infinity reference, ${\varvec{\upvarphi }}$ is the ideal EEG potential with the infinity reference, ${\mathbf{j}}$ is the equivalent neural source currents (Plonsey and Heppner 1967). The EEG potentials are the attenuated and mixed neural activities resulting from the heterogenous conductivities of head compartments, e.g. skin, skull, brain etc. The volume conduction model (1) is widely accepted in the EEG field and holds true regardless of the reference particularly adopted—changing the reference only implies modifying the lead field.

### Previous Attempts and Recent Progress

Previous notable attempts to design an optimal reference have been to carry out online recording with respect to a cephalic reference electrode e.g. Cz, Fz, Oz and FCz, etc. and subsequent offline computation of a different reference (re-referencing). Examples are such as the linked mastoids (LM) (Gibbs et al. 1936; Faux et al. 1990), the average reference (AR) (Goldman 1950; Offner 1950), the reference electrode standardization technique (REST) (Yao 2001), and its regularized version (rREST) (Hu et al. 2018c). All of these are URs (Hu et al. 2018b), meaning that all the active electrodes are referenced to a unique reference signal. By contrast, other also proposed the non-URs, such as the bipolar reference recordings (Berger 1929; Niedermeyer and Da Silva 2005) and the scalp Laplacian (Hjorth 1975; Pascual-marqui et al. 1988; Perrin et al. 1989).

It is not difficult to discern that the reference signal of online recording reference electrode or the offline re-references (LM, AR) is just a linear combination to the ideal EEG potentials ${\varvec{\upvarphi }}$ referenced at infinity. In recent years, AR and REST have been the two of the most widely adopted references. The justification of AR is that if the head is modeled as a layered spherical sphere with the neural currents spreading in a isotropic way, the discrete integral of the potentials over the head surface will be zero (Bertrand et al. 1985). REST is a method to approximately reconstruct the potentials ${\varvec{\upvarphi }}$, by making use of the forward head model and the equivalent sources model shown in (1) (Yao 2001). A more recent development is rREST which also deals with the denoising problem via the generalized cross validation criterion (Hu et al. 2018c). It is shown as well in the same paper that the use of a population average lead field yields better results than the use of spherical lead field. With this variety of URs to pick from, it is evident that a unified model is needed to analyze interrelations between reference procedures as well as to compare their properties.

### Remaining Problems and Proposals

REST is based on the fact that EEG activities are ultimately generated by the same neural sources no matter what reference is used. The introduction of REST has stimulated an increasing number of comparative studies on how different references affect experimental data analysis (Bonfiglio et al. 2013; Tian and Yao 2013; Kugiumtzis and Kimiskidis 2015; Chella et al. 2016; Mumtaz and Malik 2018). However, we felt that purely empirical comparisons may be incomplete. Several questions remained unanswered. For instances, how can one formulate in a single model all the reference transformations? What this type of model reveals about the connections among the various references? Are all the URs dependent on each other? Is REST an UR which drags along the remaining impacts of previous use of other references? What are the common properties of the URs? Is it possible to achieve the unbiased estimator to the ideal infinity reference? What are the statistical interpretations for AR and REST? Are AR and REST valid from the view of mathematical statistics? These questions came to the forefront during drafting the accompanying review of the EEG reference problem (Yao et al. 2019).

In responding to these questions, we found that UR transformations always reduce the rank by 1 of multichannel EEG potentials referenced to infinity. Thus, estimating the ideal potentials of full rank from the singular reference transforming matrix is an underdetermined or rank deficient linear regression problem (Mardia et al. 1979; Magnus and Heinz 2007). This is therefore an inverse problem of a different nature but related to the source localization. Fortunately, the required tools to attack this problem have already been developed related to Moore–Penrose pseudoinverse of modified matrices by rank one subtraction (Meyer Jr. 1973; Trenkler 2000; Baksalary et al. 2003).

In this brief communication, we propose the general form of the EEG reference problem, demonstrate that REST is a special type of UR, generalize the family of possible URs, summarize the notable properties of them and derive the AR and REST from the MLE and the Bayesian theory (Table 1).

**Table 1:** Mathematical notations

$N_{c}$	The number of channels	$N_{s}$	The number of neural sources
${\mathbb{R}}^{m \times n}$	The real matrices of $m$ rows by $n$ columns	$rk$	The matrix rank operator
$r$	The unipolar references (URs)	${\mathbf{T}}_{r} \in {\mathbb{R}}^{{N_{c} \times N_{c} }}$	The UR transforming operator
${\varvec{\upvarphi }} \in {\mathbb{R}}^{{N_{c} \times 1}}$	The instantaneous EEG potentials with infinity reference	${\mathbf{K}}_{\infty } \in {\mathbb{R}}^{{N_{c} \times N_{s} }}$	The lead field matrix with infinity reference
${\mathbf{v}}_{r} \in {\mathbb{R}}^{{N_{c} \times 1}}$	The unipolar referenced EEG potentials over all channels	${\mathbf{j}} \in {\mathbb{R}}^{{N_{s} \times 1}}$	The brain neural source currents
${\mathbf{f}}_{r} \in {\mathbb{R}}^{{N_{c} \times 1}}$	The vector of linear coefficients for URs	${\mathbf{1}} \in {\mathbb{R}}^{{N_{c} \times 1}}$	A vector of ones
${\mathbf{M}}^{ + }$	The Moore–Penrose inverse of matrix ${\mathbf{M}}$	${\mathbf{I}}_{{N_{c} }}$	The identity matrix
${\mathbf{M}}^{{\mathbf{T}}}$	The transpose operator of matrix ${\mathbf{M}}$	${\varvec{\Sigma}}$	The covariance matrix

## Demonstration

### General Form of the Reference Problem

In practice one can never observe **φ**, because the infinity reference is practically unachievable. What one observed is, instead, the referenced data ${\mathbf{x}}$, that may be either the UR recordings ${\mathbf{v}}_{r}$ or the non-UR recordings e.g. the currents by bipolar recordings and the current source density by the scalp Laplacian. Each type of referenced data is a linear transformation via pre-multiplication of the transforming matrix ${\mathbf{T}}_{\text{o}}$ with the EEG potentials **φ** plus the sensor noise ${\varvec{\upvarepsilon}}$. Thus, the general form of the reference problem is:

2 $${\mathbf{x}} = {\mathbf{T}}_{\text{o}} ({\varvec{\upvarphi }} + {\varvec{\upvarepsilon}}) = {\mathbf{T}}_{\text{o}} {\varvec{\upvarphi }} + {\varvec{\upvarepsilon}}_{ \circ }$$

where ${\mathbf{T}}_{\text{o}}$ is a non-stochastic matrix of observations, **φ** is the ideal potentials with infinity reference supposed to be a deterministic fixed but unknown vector, and ${\varvec{\upvarepsilon}}$ is the non-observable random sensor noise disturbances. Apparently the estimation to **φ** in solving the EEG reference problem (2) is an underdetermined linear regression problem.

Without loss of generality, ${\mathbf{x}}$ and ${\varvec{\upvarepsilon}}$ are considered to have the multivariate normal distribution. If the sensor noise has an independent identical distribution (IID) across channels, the covariance of sensor noise in the referenced data will be ${\varvec{\Sigma}}_{{{\varvec{\upvarepsilon}}_{\text{o}} {\varvec{\upvarepsilon}}_{\text{o}} }} = \sigma^{2} {\mathbf{T}}_{\text{o}} {\mathbf{T}}_{\text{o}}^{{\mathbf{T}}}$ because the referencing effect is taken on the noise as well during recording (Pascual-Marqui et al. 1994).

### The Family of Unipolar References (URs)

Although ${\mathbf{T}}_{\text{o}}$ can be either the 1st derivative in the bipolar recordings or the 2nd differential operator in the scalp Laplacian. Both however quantify differently the EEG signals from potentials, henceforth we will concentrate on taking ${\mathbf{T}}_{\text{o}}$ with the UR operator ${\mathbf{T}}_{r}$ and the UR transforming is thus

3 $${\mathbf{v}}_{r} = {\mathbf{T}}_{r} {\varvec{\upvarphi }} + {\varvec{\upvarepsilon}}_{r}$$

Unipolar reference (UR) is regarded if all the electrodes are referenced to a unique physical reference or a unique virtual reference. The physical reference is usually the electrode (e.g. Cz, Fz, Oz and FCz) placed on the scalp or body surface during online recording setup. The virtual reference is a linear combination of the recordings from all the electrodes, usually obtained during offline processing after the EEG data acquisition. Typical examples of virtual references are the LM, AR and REST.

The reference operator in (3) has a common structure (Hu et al. 2018b) for the family of URs as,

4 $${\mathbf{T}}_{r} = {\mathbf{I}}_{{N_{c} }} - {\mathbf{1f}}_{r}^{{\mathbf{T}}}$$

where ${\mathbf{f}}_{r}$ consists of the linear combination weights of all the electrodes. The family of URs is tabulated in the Table 2 with ${\mathbf{f}}_{r} \in \{ {\mathbf{f}}_{RR} ,{\mathbf{f}}_{LM} ,{\mathbf{f}}_{AR} ,{\mathbf{f}}_{REST} \}$. Note that the nonzero entries (i.e. 1 and 0.5) of ${\mathbf{f}}_{RR}$ and ${\mathbf{f}}_{LM}$ correspond to the indices of a unique physical reference electrode (Cz, Fz, Oz, or FCz etc.) and the two mastoids/earlobes, respectively (Hu et al. 2018b). The electrodes of two mastoids/earlobes are usually labeled as A1-A2, M1-M2, or referring to TP9 and TP10 in the fixed electrode layouts.

**Table 2:** The family of unipolar references

Unipolar references	${\mathbf{v}}_{r} = {\mathbf{T}}_{r} {\varvec{\upvarphi }} + {\varvec{\upvarepsilon}}_{r}$, ${\mathbf{T}}_{r} = {\mathbf{I}}_{{N_{c} }} - {\mathbf{1f}}_{r}^{{\mathbf{T}}}$, ${\mathbf{f}}_{r} \in \{ {\mathbf{f}}_{RR} ,{\mathbf{f}}_{LM} ,{\mathbf{f}}_{AR} ,{\mathbf{f}}_{REST} \}$	
Online recording references	Cz, Fz, Oz and FCz, etc. (RR)	${\mathbf{f}}_{RR} = [0, \ldots ,0,1,0, \ldots ,0]^{{\mathbf{T}}}$
Offline re-references	Linked mastoids (LM)	${\mathbf{f}}_{LM} = [0, \ldots ,0,0.5,0, \ldots ,0,0.5,0, \ldots 0]^{{\mathbf{T}}}$
	Average reference (AR)	${\mathbf{f}}_{AR} = {{\mathbf{1}} \mathord{\left/ {\vphantom {{\mathbf{1}} {N_{c} }}} \right. \kern-0pt} {N_{c} }}$
	REST	${\mathbf{f}}_{REST} = {{{\mathbf{K}}_{\infty }^{{ + {\mathbf{T}}}} {\mathbf{K}}_{\infty }^{ + } {\mathbf{1}}} \mathord{\left/ {\vphantom {{{\mathbf{K}}_{\infty }^{{ + {\mathbf{T}}}} {\mathbf{K}}_{\infty }^{ + } {\mathbf{1}}} {[{\mathbf{1}}^{{\mathbf{T}}} {\mathbf{K}}_{\infty }^{{ + {\mathbf{T}}}} {\mathbf{K}}_{\infty }^{ + } {\mathbf{1}}}}} \right. \kern-0pt} {[{\mathbf{1}}^{{\mathbf{T}}} {\mathbf{K}}_{\infty }^{{ + {\mathbf{T}}}} {\mathbf{K}}_{\infty }^{ + } {\mathbf{1}}}}]$

Among the family of URs, AR is one of the most widely used methods to estimate the potentials ${\varvec{\upvarphi }}$ with infinity reference as

5 $${\mathbf{T}}_{AR} = {\mathbf{I}}_{{N_{c} }} - {\mathbf{1f}}_{AR}^{{\mathbf{T}}} ,\quad {\mathbf{f}}_{AR} = {{\mathbf{1}} \mathord{\left/ {\vphantom {{\mathbf{1}} {N_{c} }}} \right. \kern-0pt} {N_{c} }}$$

It is justified that for a perfect layered spherical head, with neural currents spreading in an isotropic way, the integral of the potential over the head surface is zero (Bertrand et al. 1985; Yao 2017). Thus, the averaged potential over all electrodes may tend to zero and would be suitable as the reference signal.

REST employs the equivalent source technique to transform one reference recording to another as

6 $${\hat{\varvec{\upvarphi }}}_{REST} = {\mathbf{K}}_{\infty } ({\mathbf{K}}_{r}^{ + } {\mathbf{v}}_{r} ) = ({\mathbf{K}}_{\infty } {\mathbf{K}}_{r}^{ + } ){\mathbf{v}}_{r} = {\mathbf{R}}_{r} {\mathbf{v}}_{r}$$

where ${\mathbf{R}}_{r} = {\mathbf{K}}_{\infty } {\mathbf{K}}_{r}^{ + }$ is the reference standardization matrix depending on the reference ${\mathbf{T}}_{r}$ implicitly ‘embedded’ in the EEG data ${\mathbf{v}}_{r}$, and the equivalent source is approximately estimated as ${\hat{\mathbf{j}}} = {\mathbf{K}}_{r}^{ + } {\mathbf{v}}_{r}$ (Yao 2001). Since ${\mathbf{R}}_{r}$ is transforming the referenced data ${\mathbf{v}}_{r}$, REST was described as a transformation of data already subject to a previous reference such as AR. This was in apparent contrast to LM and AR which both transform the ideal potentials ${\varvec{\upvarphi }}$ at infinity. To allow a closer look of REST, it requires an explicit expression on how it transforms the ideal potentials ${\varvec{\upvarphi }}$ at infinity (Hu et al. 2018b). The unipolar form of ${\mathbf{T}}_{REST}$ is derived next.

### Demonstration of REST as an UR

The REST operator is defined as

7 $${\mathbf{T}}_{REST} = {\mathbf{K}}_{\infty } {\mathbf{K}}_{r}^{ + } {\mathbf{T}}_{r}$$

by post-multiplying ${\mathbf{R}}_{r}$ with the reference ${\mathbf{T}}_{r}$ hidden in the data ${\mathbf{v}}_{r}$ (Hu et al. 2018b). The lead field referenced to the same UR as (4) is

8 $${\mathbf{K}}_{r} = {\mathbf{T}}_{r} {\mathbf{K}}_{\infty } = {\mathbf{K}}_{\infty } + {\mathbf{1}}( - {\mathbf{K}}_{\infty }^{{\mathbf{T}}} {\mathbf{f}}_{r} )^{{\mathbf{T}}}$$

Since the number of distributed neural sources is much larger than the number of electrodes and because of the volume conductivities, ${\mathbf{K}}_{\infty }$ has all independent rows namely full row rank, leading to ${\mathbf{K}}_{\infty } {\mathbf{K}}_{\infty }^{ + } = {\mathbf{I}}_{{N_{c} }}$ and $rk({\mathbf{K}}_{r} ) = rk({\mathbf{T}}_{r} )$. Noting that ${\mathbf{T}}_{r}$ is with full rank deficient by 1 (Hu et al. 2018c), thus $rk({\mathbf{K}}_{r} ) = rk({\mathbf{K}}_{\infty } ) - 1$ which is the case (↓) of the Theorem 1.1 in (Baksalary et al. 2003). By defining ${\mathbf{d}} = - {\mathbf{K}}_{\infty }^{ + } {\mathbf{1}}$ as the Formula (1.3) in (Baksalary et al. 2003), we have

9 $${\mathbf{K}}_{r}^{ + } {\mathbf{K}}_{r} = {\mathbf{K}}_{\infty } {\mathbf{K}}_{\infty }^{ + } - \frac{{{\mathbf{dd}}^{{\mathbf{T}}} }}{{{\mathbf{d}}^{{\mathbf{T}}} {\mathbf{d}}}} = {\mathbf{I}}_{{N_{c} }} - \frac{{{\mathbf{K}}_{\infty }^{ + } {\mathbf{11}}^{{\mathbf{T}}} {\mathbf{K}}_{\infty }^{{ + {\mathbf{T}}}} }}{{{\mathbf{1}}^{{\mathbf{T}}} {\mathbf{K}}_{\infty }^{{ + {\mathbf{T}}}} {\mathbf{K}}_{\infty }^{ + } {\mathbf{1}}}}$$

according to the case (↓) in the list 2.2 of the Theorem 2.1 (Baksalary et al. 2003).

Post-multiplying ${\mathbf{K}}_{\infty } {\mathbf{K}}_{\infty }^{ + } = {\mathbf{I}}_{{N_{c} }}$, the REST operator in (7) is equivalent to

10 $${\mathbf{T}}_{REST} = {\mathbf{K}}_{\infty } {\mathbf{K}}_{r}^{ + } {\mathbf{K}}_{r} {\mathbf{K}}_{\infty }^{ + } = {\mathbf{I}}_{{N_{c} }} - {\mathbf{1}}\frac{{{\mathbf{1}}^{{\mathbf{T}}} {\mathbf{K}}_{\infty }^{{ + {\mathbf{T}}}} {\mathbf{K}}_{\infty }^{ + } }}{{{\mathbf{1}}^{{\mathbf{T}}} {\mathbf{K}}_{\infty }^{{ + {\mathbf{T}}}} {\mathbf{K}}_{\infty }^{ + } {\mathbf{1}}}}$$

Obviously, REST operator belongs to the family of URs. Written as ${\mathbf{T}}_{REST} = {\mathbf{I}}_{{N_{c} }} - {\mathbf{1f}}_{REST}^{{\mathbf{T}}}$, the linear combination weights for REST is

11 $${\mathbf{f}}_{REST} = {{{\mathbf{K}}_{\infty }^{{ + {\mathbf{T}}}} {\mathbf{K}}_{\infty }^{ + } {\mathbf{1}}} \mathord{\left/ {\vphantom {{{\mathbf{K}}_{\infty }^{{ + {\mathbf{T}}}} {\mathbf{K}}_{\infty }^{ + } {\mathbf{1}}} {[{\mathbf{1}}^{{\mathbf{T}}} {\mathbf{K}}_{\infty }^{{ + {\mathbf{T}}}} {\mathbf{K}}_{\infty }^{ + } {\mathbf{1}}]}}} \right. \kern-0pt} {[{\mathbf{1}}^{{\mathbf{T}}} {\mathbf{K}}_{\infty }^{{ + {\mathbf{T}}}} {\mathbf{K}}_{\infty }^{ + } {\mathbf{1}}]}}$$

Therefore, REST operator admits the same form of URs defined in (4). While the reference standardization matrix ${\mathbf{R}}_{r}$ is dependent on the prior EEG reference, the REST operator ${\mathbf{T}}_{REST}$ is independent of the specific UR in the EEG data and will be identical whichever ${\mathbf{T}}_{r}$ is adopted in (7) noting that ${\mathbf{f}}_{r}$ disappears in (11). The demonstration of REST as a UR clarifies its relation to other references.

### Properties of URs: No Memory, Rank Deficient by 1 and Orthogonal Projector Centering

We analyzed the family of URs and naturally found some valuable properties of URs summarized as ‘no memory’, ‘rank deficient by 1’ and ‘orthogonal projector centering’.

(1) No memory property

Supposing ${\mathbf{T}}_{r1} = {\mathbf{I}}_{{N_{c} }} - {\mathbf{1f}}_{r1}^{{\mathbf{T}}}$ is the latest reference one intends to apply, and ${\mathbf{T}}_{r2}$ is the previous reference already applied in the EEG data, as long as ${\mathbf{f}}_{r1}^{{\mathbf{T}}} {\mathbf{1}} = 1$, one will have

12 $${\mathbf{T}}_{r1} = {\mathbf{T}}_{r1} {\mathbf{T}}_{r2}$$

where ${\mathbf{T}}_{r2}$ could be any UR operator.

Note that ${\mathbf{f}}_{r}^{{\mathbf{T}}} {\mathbf{1}} = 1$ for ${\mathbf{f}}_{r} \in \{ {\mathbf{f}}_{RR} ,{\mathbf{f}}_{LM} ,{\mathbf{f}}_{AR} ,{\mathbf{f}}_{REST} \}$, this no memory property holds true for the family of URs including both online recording references e.g. Cz, Fz, Oz and FCz, etc. and the offline re-references such as LM, AR and REST.

(2) Rank deficient by 1 property

For the URs ${\mathbf{T}}_{r}$ with ${\mathbf{f}}_{r} \in \{ {\mathbf{f}}_{RR} ,{\mathbf{f}}_{LM} ,{\mathbf{f}}_{AR} ,{\mathbf{f}}_{REST} \}$, it is found

13 $$rk({\mathbf{T}}_{r} ) = N_{c} - 1$$

which means the rank of ${\mathbf{T}}_{r}$ are all full rank deficient by 1.

(3) Orthogonal projector centering property

The orthogonal projector onto the column space of ${\mathbf{T}}_{r}^{{\mathbf{T}}}$ as the centering matrix (i.e. the averager reference)

14 $${\mathbf{T}}_{r}^{ + } {\mathbf{T}}_{r} = {\mathbf{T}}_{AR}$$

The readers can refer to the appendix of (Hu et al. 2018c) for the proofs of ‘rank deficient by 1’ and ‘orthogonal projector centering’ properties.

From the “Demonstration of REST as an UR” section, the lead field matrices also have the ‘identity’ property ${\mathbf{K}}_{\infty } {\mathbf{K}}_{\infty }^{ + } = {\mathbf{I}}_{{N_{c} }}$, the ‘rank deficient by 1’ property $rk({\mathbf{K}}_{r} ) = rk({\mathbf{K}}_{\infty } ) - 1$, and the ‘orthogonal projector weighted centering’ property ${\mathbf{K}}_{r}^{ + } {\mathbf{K}}_{r} = {\mathbf{I}}_{{N_{c} }} - {\mathbf{K}}_{\infty }^{ + } {\mathbf{11}}^{{\mathbf{T}}} {\mathbf{K}}_{\infty }^{{ + {\mathbf{T}}}} /{\mathbf{1}}^{{\mathbf{T}}} {\mathbf{K}}_{\infty }^{{ + {\mathbf{T}}}} {\mathbf{K}}_{\infty }^{ + } {\mathbf{1}}$. Note that the ‘rank deficient by 1’ and ‘orthogonal projector centering’ properties of the URs follow trivially if the lead field matrix ${\mathbf{K}}_{\infty }$ is an identity matrix. However, realistic lead fields ${\mathbf{K}}_{\infty }$ are far from the identity. The biophysical assumptions are the cause of the difference of REST from the other URs.

### Derivation of AR and REST from the Maximum Likelihood Estimate

The actual purpose in searching for the best reference is to estimate potentials at infinity rather than to identify the reference signal. The unipolar reference model in (3) is written as the block form

15 $$\left[ {\begin{array}{*{20}l} {{\mathbf{v}}_{r - } } \hfill \\ {v_{r} } \hfill \\ \end{array} } \right] = \left[ {\begin{array}{*{20}l} {{\mathbf{T}}_{r - } } \hfill \\ {{\mathbf{t}}_{r}^{{\mathbf{T}}} } \hfill \\ \end{array} } \right]{\varvec{\upvarphi }} + {\mathbf{\upvarepsilon}}_{r}$$

where ${\mathbf{T}}_{r - } \in {\mathbb{R}}^{{(N_{c} - 1) \times N_{c} }}$ is a ‘fat’ matrix, ${\mathbf{t}}_{r} \in {\mathbb{R}}^{{N_{c} \times 1}}$, ${\mathbf{v}}_{r - } \in {\mathbb{R}}^{{(N_{c} - 1) \times 1}}$ are vectors, and $v_{r}$ is a scalar.

Since ${\mathbf{T}}_{r}$ is rank deficient by 1, discarding one row yields a matrix of full row rank. Specifically, if ${\mathbf{T}}_{r}$ is the recording reference, ${\mathbf{t}}_{r}$ corresponds to the physical reference electrode; if ${\mathbf{T}}_{r}$ is the liked mastoids/earlobes reference, ${\mathbf{t}}_{r}$ corresponds to either of the electrodes at two mastoids/earlobes. Thus, the UR model reduces to

16 $${\mathbf{v}}_{r - } = {\mathbf{T}}_{r - } {\varvec{\upvarphi }} + {\varvec{\upvarepsilon}}_{r - }$$

where the covariance of ${\varvec{\upvarepsilon}}_{r - }$ is ${\varvec{\Sigma}}_{{{\varvec{\upvarepsilon}}_{r - } {\varvec{\upvarepsilon}}_{r - } }} = \sigma^{2} {\mathbf{T}}_{r - } {\mathbf{T}}_{r - }^{{\mathbf{T}}}$.

It is apparent that without the constraint, estimating ${\varvec{\upvarphi }} \in R^{{N_{c} \times 1}}$ from ${\mathbf{v}}_{r - } \in R^{{(N_{c} - 1) \times 1}}$ is underdetermined.

The constraint of the average reference (AR) is

17 $${\mathbf{1}}^{{\mathbf{T}}} {\varvec{\upvarphi }} = 0$$

the physical meaning of which is the discrete integral as zero of electric potentials over a layered spherical and isotropic conductor surface (Bertrand et al. 1985). The estimation to ${\varvec{\upvarphi }}$ is a linear regression problem with this constraint. Making use of theorem 6 in the page 303 of (Magnus and Heinz 2007), the best linear unbiased estimator to (16) is

18 $${\hat{\varvec{\upvarphi }}} = \left( {{\mathbf{I}} - {{{\mathbf{P}}^{ - 1} {\mathbf{11}}^{{\mathbf{T}}} } \mathord{\left/ {\vphantom {{{\mathbf{P}}^{ - 1} {\mathbf{11}}^{{\mathbf{T}}} } {{\mathbf{1}}^{{\mathbf{T}}} {\mathbf{P}}^{ - 1} {\mathbf{1}}}}} \right. \kern-0pt} {{\mathbf{1}}^{{\mathbf{T}}} {\mathbf{P}}^{ - 1} {\mathbf{1}}}}} \right){\mathbf{P}}^{ - 1} {\mathbf{T}}_{r - }^{{\mathbf{T}}} ({\mathbf{T}}_{r - } {\mathbf{T}}_{r - }^{{\mathbf{T}}} )^{ - 1} {\mathbf{v}}_{r - }$$

with ${\mathbf{P}} = {\mathbf{T}}_{r - }^{{\mathbf{T}}} ({\mathbf{T}}_{r - } {\mathbf{T}}_{r - }^{{\mathbf{T}}} )^{ - 1} {\mathbf{T}}_{r - } + {\mathbf{11}}^{{\mathbf{T}}}$. Due to ${\mathbf{T}}_{r - }^{{\mathbf{T}}} ({\mathbf{T}}_{r - } {\mathbf{T}}_{r - }^{{\mathbf{T}}} )^{ - 1} = {\mathbf{T}}_{r - }^{ + }$ and ${\mathbf{T}}_{r - }^{ + } {\mathbf{T}}_{r - } = {\mathbf{T}}_{AR}$, ${\mathbf{P}}$ is written as

$${\mathbf{P}} = {\mathbf{I}} + {\mathbf{11}}^{{\mathbf{T}}} /[{\mathbf{1}}^{{\mathbf{T}}} {\mathbf{1}}/({\mathbf{1}}^{{\mathbf{T}}} {\mathbf{1}} - 1)]$$

And its inverse is solved by the Formula (2.2) in (Baksalary et al. 2003) as

19 $${\mathbf{P}}^{ - 1} = {{{\mathbf{I}} - ({\mathbf{1}}^{{\mathbf{T}}} {\mathbf{1}} - 1){\mathbf{11}}^{{\mathbf{T}}} } \mathord{\left/ {\vphantom {{{\mathbf{I}} - ({\mathbf{1}}^{{\mathbf{T}}} {\mathbf{1}} - 1){\mathbf{11}}^{{\mathbf{T}}} } {{\mathbf{1}}^{{\mathbf{T}}} {\mathbf{11}}^{{\mathbf{T}}} {\mathbf{1}}}}} \right. \kern-0pt} {{\mathbf{1}}^{{\mathbf{T}}} {\mathbf{11}}^{{\mathbf{T}}} {\mathbf{1}}}}$$

Substituting (19) into (18), it is simplified as

20 $${\hat{\varvec{\upvarphi }}} = {\mathbf{T}}_{AR} {\mathbf{T}}_{r - }^{ + } {\mathbf{v}}_{r - } = {\mathbf{T}}_{AR} {\mathbf{T}}_{r - }^{ + } ({\mathbf{T}}_{r - } {\varvec{\upvarphi }} + {\mathbf{\upvarepsilon}}_{r - } ) = {\mathbf{T}}_{AR} ({\varvec{\upvarphi }} + {\mathbf{\upvarepsilon}})$$

which is the AR if the sensor noise tends to zero or one neglects the noise. This shows that AR can be derived by constraining the sum over all electrodes as zero and the best linear unbiased estimator to the infinity reference would be the AR if the given constraint holds true and sensor noise is negligible.

In the case of REST, taking the singular value decomposition (SVD) of the lead field as ${\mathbf{K}}_{\infty } = {\mathbf{USW}}^{{\mathbf{T}}}$, (16) is expressed as

21 $${\mathbf{v}}_{r - } = {\mathbf{T}}_{r - } {\mathbf{USW}}^{{\mathbf{T}}} {\mathbf{j}} + {\varvec{\upvarepsilon}}_{r - }$$

Defining ${\mathbf{L}} = {\mathbf{T}}_{r - } {\mathbf{US}}$ and ${\mathbf{\upbeta}} = {\mathbf{W}}^{{\mathbf{T}}} {\mathbf{j}}$, (21) is

22 $${\mathbf{v}}_{r - } = {\mathbf{L}}{\mathbf{\upbeta}} + {\mathbf{T}}_{r - } {\varvec{\upvarepsilon}}$$

The constraint for REST is

23 $$\hbox{min} \left\| {\varvec{\upbeta}} \right\|_{M}^{2}$$

where $M$ means the Mahalanobis distance. This means REST doesn’t depend on the particular inverse solution but rather on the parameter ${\mathbf{\upbeta}} = {\mathbf{W}}^{{\mathbf{T}}} {\mathbf{j}}$. The constraint REST poses is to minimize the term structured by the forward model (lead field) and true neural sources. However, since ${\mathbf{W}}$ is an orthonormal matrix, the minimum norm of ${\mathbf{\upbeta}}$ equals to the minimum Euclidean norm of ${\mathbf{j}}$ when the neural source ${\mathbf{j}}$ has a priori IID covariance.

Solving (23) but subject to (22), it is

24 $${\hat{\mathbf{\beta }}} = {\varvec{\Sigma}}_{{{\varvec{\upbeta \upbeta }}}} {\mathbf{L}}^{{\mathbf{T}}} ({\mathbf{L\varSigma }}_{{{\varvec{\upbeta \upbeta }}}} {\mathbf{L}}^{{\mathbf{T}}} + {\mathbf{T}}_{r - } {\varvec{\Sigma}}_{{{\varvec{\upvarepsilon \upvarepsilon }}}} {\mathbf{T}}_{r - }^{{\mathbf{T}}} )^{ - 1} {\mathbf{v}}_{r - }$$

Taking the equivalent source ${\varvec{\Sigma}}_{{{\mathbf{jj}}}} = \alpha^{2} {\mathbf{I}}_{{N_{s} }}$ and given ${\mathbf{K}}_{r - } = {\mathbf{T}}_{r - } {\mathbf{USW}}^{{\mathbf{T}}}$, ${\mathbf{K}}_{\infty } {\mathbf{K}}_{\infty }^{{\mathbf{T}}} = {\mathbf{US}}^{2} {\mathbf{U}}^{{\mathbf{T}}}$, (24) multiplicated with ${\mathbf{US}}$ becomes

$${\hat{\varvec{\upvarphi }}} = {\mathbf{K}}_{\infty } \cdot {\mathbf{K}}_{r - }^{{\mathbf{T}}} \left( {{\mathbf{K}}_{r - } {\mathbf{K}}_{r - }^{{\mathbf{T}}} + \frac{{\sigma^{2} }}{{\alpha^{2} }}{\mathbf{T}}_{r - } {\mathbf{T}}_{r - }^{{\mathbf{T}}} } \right)^{ - 1} {\mathbf{v}}_{r - }$$

when $\sigma$ tends to zero or the sensor noise is neglected, ${\hat{\varvec{\upvarphi }}} = {\mathbf{K}}_{\infty } {\mathbf{K}}_{r - }^{ + } {\mathbf{v}}_{r - }$ becomes REST. REST assumes potentials generated by a lead field for which a minimum norm constraint may be imposed.

Note that ${\mathbf{K}}_{\infty }$ is with one additional channel to ${\mathbf{K}}_{r - }$ indicating the interpolation function of REST. This function can recover the full recordings over all channels from the recordings with the reference channel missing or even be generalized to interpolate bad channels that have been rejected.

### Derivation of AR and REST from the Bayesian Theory

All references are the linear combination of the ideal potentials with infinity reference. It turns a linear transformation through the lead field of actual neural source activity. Therefore estimating ${\hat{\varvec{\upvarphi }}}_{\text{o}}$ is the solution to a linear undetermined inverse problem. And it is essential to work with the brain sources. Any estimator of the potentials at infinity is the maximum a posterior (MAP) estimator with the Bayesian theory

25 $$p({\varvec{\upvarphi }}\left| {{\mathbf{x}},{\mathbf{T}}_{\text{o}} ,{\varvec{\upvarepsilon}}} \right.) \propto p({\mathbf{x}}\left| {{\varvec{\upvarphi }},{\mathbf{T}}_{\text{o}} ,{\varvec{\upvarepsilon}}} \right.)p({\varvec{\upvarphi }})p({\mathbf{\upvarepsilon}})$$

where $p({\varvec{\upvarphi }}\left| {{\mathbf{x}},{\mathbf{T}}_{\text{o}} ,{\varvec{\upvarepsilon}}} \right.)$ is the posterior given the likelihood $p({\mathbf{x}}\left| {{\varvec{\upvarphi }},{\mathbf{T}}_{\text{o}} ,{\varvec{\upvarepsilon}}} \right.)$ and priors $p({\varvec{\upvarphi }})$,$p({\varvec{\upvarepsilon}})$. With the tuning parameter $\lambda$, (25) can be converted by (Mardia et al. 1979) into

26 $$\ell = \left\| {{\mathbf{x}} - {\mathbf{T}}_{\text{o}} {\varvec{\upvarphi }}} \right\|_{M}^{2} + \lambda \left\| {\varvec{\upvarphi }} \right\|_{M}^{2}$$

Solving the formula (26) derives the MAP estimator to the potentials at infinity as

27 $${\hat{\varvec{\upvarphi }}}_{\text{o}} = {\varvec{\Sigma}}_{{{\mathbf{\varphi \varphi }}}} {\mathbf{T}}_{\text{o}}^{{\mathbf{T}}} ({\mathbf{T}}_{\text{o}} {\varvec{\Sigma}}_{{{\mathbf{\varphi \varphi }}}} {\mathbf{T}}_{\text{o}}^{{\mathbf{T}}} + \sigma^{2} {\mathbf{T}}_{\text{o}} {\mathbf{T}}_{\text{o}}^{{\mathbf{T}}} )^{ + } {\mathbf{x}}$$

which is the general solution to the EEG reference problem using the Bayesian theory. Specifying the reference transformation matrix ${\mathbf{T}}_{\text{o}}$ as ${\mathbf{T}}_{r}$, the difference between ${\hat{\varvec{\upvarphi }}}_{\text{o}}$ estimators is only in the prior covariance ${\varvec{\Sigma}}_{{{\mathbf{\varphi \varphi }}}}$ assumed for the potentials at infinity.

AR is the special case of (27) with the IID prior ${\varvec{\Sigma}}_{{{\mathbf{\varphi \varphi }}}} = \alpha^{2} {\mathbf{I}}_{{N_{c} }}$. This results in the minimum norm least square solution:

28 $${\hat{\varvec{\upvarphi }}}_{r} = \alpha^{2} {\mathbf{T}}_{r}^{{\mathbf{T}}} (\alpha^{2} {\mathbf{T}}_{r} {\mathbf{T}}_{r}^{{\mathbf{T}}} + \sigma^{2} {\mathbf{T}}_{r} {\mathbf{T}}_{r}^{{\mathbf{T}}} )^{ + } {\mathbf{v}}_{r}$$

When $\sigma^{2}$ tends to zero and substituting (3) into (28), this expression simplifies to

29 $${\hat{\varvec{\upvarphi }}}_{{_{r} }} = {\mathbf{T}}_{r}^{ + } {\mathbf{T}}_{r} {\varvec{\upvarphi }} + {\mathbf{T}}_{r}^{ + } {\varvec{\upvarepsilon}}_{r}$$

Since the ‘orthogonal projector centering’ property holds true for the family of URs, thus

30 $${\hat{\varvec{\upvarphi }}}_{AR} = {\hat{\varvec{\upvarphi }}}_{r} = {\mathbf{T}}_{AR} {\varvec{\upvarphi }} + {\varvec{\upvarepsilon}}_{AR}$$

Hence, when the priori covariance of ${\varvec{\upvarphi }}$ is IID, the minimum norm solution of (3) with any UR is same as the AR. It also confirms that AR can only be applied to the EEG data that has already been transformed by the other URs (Hu et al. 2018a).

By contrast, REST is derived if the potentials ${\varvec{\upvarphi }}$ with infinity reference are considered to be generated by the neural sources with a priori IID covariance. The following version of (2) is valid for data with any reference,

31 $${\mathbf{x}} = {\mathbf{T}}_{\text{o}} {\varvec{\upvarphi }} + {\varvec{\upvarepsilon}}_{\text{o}} = {\mathbf{K}}_{\text{o}} {\mathbf{j}} + {\varvec{\upvarepsilon}}_{\text{o}}$$

where ${\mathbf{K}}_{\text{o}} = {\mathbf{T}}_{\text{o}} {\mathbf{K}}_{\infty }$ is the transformed forward model. From (2), with the covariance ${\varvec{\Sigma}}_{{{\mathbf{jj}}}}$ of the equivalent source, the solution to (31) is expressed as

32 $${\hat{\varvec{\upvarphi }}}_{rREST} = {\mathbf{K}}_{\infty } {\varvec{\Sigma}}_{{{\mathbf{jj}}}} {\mathbf{K}}_{\text{o}}^{{\mathbf{T}}} ({\mathbf{K}}_{\text{o}} {\varvec{\Sigma}}_{{{\mathbf{ss}}}} {\mathbf{K}}_{\text{o}}^{{\mathbf{T}}} + \sigma^{2} {\mathbf{T}}_{r} {\mathbf{T}}_{r}^{{\mathbf{T}}} )^{ + } {\mathbf{x}}$$

This is the regularized version of REST (rREST) (Hu et al. 2018c). If assuming the equivalent source are IID with the covariance ${\varvec{\Sigma}}_{{{\mathbf{ss}}}} = \alpha^{2} {\mathbf{I}}_{{N_{s} }}$, the rREST operator reduces to

33 $${\mathbf{T}}_{rREST} = \alpha^{2} {\mathbf{K}}_{\infty } {\mathbf{K}}_{ \circ }^{{\mathbf{T}}} (a^{2} {\mathbf{K}}_{ \circ } {\mathbf{K}}_{ \circ }^{{\mathbf{T}}} + \sigma^{2} {\mathbf{T}}_{r} {\mathbf{T}}_{r}^{{\mathbf{T}}} )^{ + }$$

With ${\mathbf{x}}$ referenced by the URs $r$ and $\sigma^{2}$ tends to 0 (noise free data), (33) turns as the classical REST (Yao 2001)

34 $${\hat{\varvec{\upvarphi }}}_{REST} = {\mathbf{K}}_{\infty } {\mathbf{K}}_{r}^{ + } {\mathbf{v}}_{r}$$

The REST assumes potentials generated by the Bayesian theory with the assumption of independent neural sources (Hu et al. 2018c). The influence of other covariance matrices for neural sources on REST type estimators is under study.

## Discussion

In this brief communication, the general form of the EEG reference electrodes problem is understood as a linear transformation to the potentials ${\varvec{\upvarphi }}$ referenced at infinity, that maybe either the URs or non-URs e.g. the bipolar recordings and the scalp Laplacian; the common structure of URs is recognized with unified notations; it is the first time to show the interpolation function in solving the reference problem and demonstrate REST as an UR. This allows us to study a generalized family of URs. Also, valuable properties of the URs family are summarized to establish the interrelations.

The most surprising property is ‘no memory’ indicating that URs works independently without the consequences of the URs already applied. It also means one can always safely re-reference the EEG/ERP recordings with different URs but not worry about if re-referencing multiple times will accumulate artifacts. Any two of URs can be transformed to each other and all the URs are taking effect independently. Before applying a different UR, one had better check if the present data is with UR. Transforming from non-UR to UR will damage the dataset though it is no problem to transform the data within the family of URs. This property validates that an UR can erase the effects of the other URs and it is therefore safe to apply UR multiple times. The ‘no memory’ property deserves to be kept in mind in the data preprocessing by EEG-ERP researchers.

The significant property of ‘rank deficient by 1’ implies that the URs always reduce the full rank by 1 of the ideal EEG potentials referenced at infinity. The lost one rank is because the reference signal subtracted from all the channels is a linear combination from the activities over all the channels referenced to infinity. This property tells us the impossibility of obtaining the unbiased estimator to the ideal EEG potentials ${\varvec{\upvarphi }}$ if no extra constraint is introduced. However, different URs indeed have different biases, relying on how much prior information is incorporated on estimating the ideal potentials ${\varvec{\upvarphi }}$ referenced at infinity.

The nontrivial property of ‘orthogonal projector centering’ as the AR is proved from theorem of Moore–Penrose pseudoinverse in the case of rank one modification. This property is made full use of in the derivation of AR from both the MLE and the Bayesian theory. Similarly, the lead fields engaged in REST has the properties of ‘orthogonal projector weighted centering’. Together with ‘identity’ and ‘rank deficient by 1’ properties, REST is demonstrated as an UR. It is by means of this ‘orthogonal projector (weighted) centering’ property that demonstrates that AR and REST does not depend any specific UR previously applied on the EEG data and both AR and REST can be finally derived.

Among the family of URs, AR and REST are the two main contenders. This communication shows that both the MLE and the Bayesian theory approaches allow the derivations of the AR and REST estimators. One approach is to derive AR and REST via MLE with linear and quadratic constraints respectively as general linear regression model (Magnus and Heinz 2007). AR is the best unbiased linear estimator given the linear constraint that the sum of the EEG potentials over all the channels is zero. By contrast, REST minimizes the quadratic constraint of a linear combination of equivalent sources. An alternative and more flexible approach follows from the Bayesian theory. AR was derived by assuming a priori IID covariance of multichannel EEG recordings; and REST was derived from the volume conduction and a priori IID covariance of neural sources activities.

From the view of MLE, AR is theoretically correct if the constraint of discrete integral of potentials as zero holds true. This is valid no matter which UR one starts from and the best unbiased estimator would be AR. The integral of potentials partly relies on the electrodes coverage and density (Bertrand et al. 1985; Nunez 2010). However, a recent work showed that the performance of AR is not closely related to the electrode density which is different from the common understanding to AR based on its zero integral assumption and coverage is a more important factor than the electrode density (Hu et al. 2018b). From the view of the Bayesian theory, the AR is essentially solving a generalized linear inverse problem to estimate the potentials at infinity. With the prior assumption of statistical IID across multichannel recordings, whatever the UR is, the minimum norm estimator will be the AR. The priori of IID covariance of potentials referenced to infinity is surely false, since the volume conduction effect is neglected. Also, one has to be sure the EEG data at hand is with UR before applying AR, limiting its ability in the general use (Hu et al. 2018a).

From the view of MLE, the quadratic constraint indicates that REST does not fully depend on the source configuration but rather on the effect it produces at the scalp by the equivalent source that represents infinite source configurations. When one assumes that EEG data are generated by brain sources, then in theory rREST (REST) is an optimal estimate of the potentials at infinity. REST allows the possibility that the number of channels in forward calculations can be more than those in estimating the equivalent source. With the additional channels, channels missing or rejected as ‘bad’ can be recovered with the interpolation function of REST.

It is worthwhile to emphasize that from the view of Bayesian theory, in REST the source distribution only enters the estimation as a specification of the prior; and REST is the MAP estimator for which the equivalent source approach offers the protection against sources mis-localization. The goal of REST is not to find the actual sources which are not actually necessary. rREST as the extension of REST has the ability of general application. The Eqs. (31)–(33) is rather general irrespective of which type of ${\mathbf{T}}_{\text{o}}$ applied to ${\mathbf{x}}$, meaning that the rREST can adapt to the non-UR recordings e.g. bipolar recordings, and scalp Laplacian as well.

As shown in (Hu et al. 2018c), two possible limitations of REST are the assumptions of noiseless data and the use of a spherical head model. A study with simulation and large dataset of EEG recordings was carried out, showing that unless the EEG data is with extremely high SNR, REST with spherical lead field can be used without the expense of building the realistic head models. Alternatively, an averaged lead field over a population of samples and denoising by the criterion of the generalized cross validation (GCV) should be used in the rREST practice (Hu et al. 2018c). The readers can refer to https://github.com/ShiangHu/LeadField-Pipeline for the easy runnable pipeline of the realistic head models and https://github.com/ShiangHu/Unified-EEG-reference-rREST for the use of rREST.

To conclude, this brief demonstration shows that all the common references including REST can be formulated into a family of URs with unified notations, some properties of URs e.g. ‘no memory’, ‘rank deficient by 1’ and ‘orthogonal projector centering’ may be valuable on helping the reference practice, both the MLE and the Bayesian theory can derive the AR and REST, and together with the interpolation function of REST providing the novel understanding and the statistical evidences for their use in the future EEG and ERP practice.
